# Social Media’s Impact on Public Awareness of the Effects of Dietary Habits and Fluid Consumption on Kidney Stone Formation: A Cross-Sectional Study

**DOI:** 10.3390/healthcare13212795

**Published:** 2025-11-04

**Authors:** Mansour Alnazari, Omar Ayidh Alotaibi, Abdulaziz Ali Alharbi, Saad Mohammed Alharthi, Ahmed H. Al-wadani, Muteb Obaid Alharthi, Bassam Abdulaziz Alosaimi, Abdulaziz Mohammed Alrasheed, Suliman Ahmed Albedaiwi, Turki Dibas Alharbi, Shahad Adel Alhemaid, Huda Yousef Alhashem, Wesam Khan, Emad Rajih

**Affiliations:** 1 General and Specialized Surgery Department, College of Medicine, Taibah University, Madinah 42361, Saudi Arabia; mmnazari@taibahu.edu.sa (M.A.); remad@taibahu.edu.sa (E.R.); 2 College of Medicine, Shaqra University, Dawadmi 34471, Saudi Arabia; s443420077@std.su.edu.sa (A.A.A.); ahmed.h.alwadani@gmail.com (A.H.A.-w.); s442420155@std.su.edu.sa (M.O.A.); bsam.al3teby@gmail.com (B.A.A.); abdulazizalr20@gmail.com (A.M.A.); xrblender33@gmail.com (S.A.A.); 3 College of Medicine, Bishah University, Bisha 67614, Saudi Arabia; e12344321es@gmail.com; 4 College of Medicine, Taibah University, Medina 71491, Saudi Arabia; turkialhrbi2001@gmail.com; 5 College of Medicine, Vision Colleges, Riyadh 13226, Saudi Arabia; shahad.alhemaid@gmail.com (S.A.A.); huda08041@gmail.com (H.Y.A.); 6 Department of Surgery, Faculty of Medicine, University of Tabuk, Tabuk 71491, Saudi Arabia; wkhan@ut.edu.sa

**Keywords:** kidney stone, public health, social media, dietary habits and fluid consumption, mediating effect, cross-sectional study

## Abstract

**Background**: Renal stone disease is a common urological condition considered to be greatly affected by lifestyle, dietary practices, and hydration status. With the rapid advancement and remarkable rise in digital communication, social media has become an important source of health information. However, little is known about its effects on raising public awareness of dietary and fluid-related risk factors for kidney stone formation, particularly in Middle Eastern populations. Aim: We aimed to evaluate the impact of social media platforms on public awareness of dietary habits and fluid consumption in relation to kidney stone prevention. **Methods**: A cross-sectional survey was applied to 980 adults with varying demographic characteristics. Data on social media use, dietary and fluid knowledge, and attitudes toward kidney stone prevention were collected through structured questionnaires. Statistical analyses, including regression and mediation models, were employed to identify predictors of awareness and explore pathways linking social media use to knowledge and attitudes. **Results**: Among the 980 participants (mean age = 29.9 ± 11 years; 55.4% males), 69.9% held university degrees, and 7.2% had a history of kidney stones. The overall awareness of kidney stone prevention varied, with most of the participants recognizing the protective role of adequate hydration (67%) and the adverse impact of soft-drink consumption (73.2%), while knowledge of dietary contributors such as animal protein and tea was limited. Greater knowledge and more appropriate attitudes were associated with older age, female gender, following healthcare professionals, and engagement with medical websites, YouTube, and TikTok. Mediation analysis revealed that social media influenced awareness indirectly through improvements in knowledge and attitudes. **Conclusions**: This study reveals that the digital environment shapes both public knowledge of and attitudes toward kidney stone prevention, though critical knowledge gaps persist regarding complex dietary factors. Mediation analysis indicated that the digital influence is likely channeled through improvements in knowledge and attitudes. We emphasize that source credibility is paramount; relying on official medical websites and following health professionals were the most effective strategies for boosting awareness. Therefore, expert-led educational strategies must be integrated into public health protocols.

## 1. Introduction

Kidney stones, also known as nephrolithiasis, are crystal depositions within the kidney that are usually evacuated through the urethra without causing pain. However, larger calculi may cause significant pain and require medical intervention [1]. Usually, the type of stone gets its name from its mineral makeup. Calcium is the most common mineral found in kidney stones worldwide [2]. Urolithiasis can be caused by a variety of risk factors, including age, gender, ethnicity, local climate, eating habits, physical activity, and work. Comorbidities like diabetes, high blood pressure, and obesity are also significant [3]. Affliction with renal stones is the most prevalent illness in the urinary tract system and constitutes a significant public health issue. In addition, urinary tract stones are a significant public health concern in Saudi Arabia, where they are common (20%). Furthermore, they tend to be more common in men than in women, with a ratio of 3.2:1, and most of the cases are idiopathic, with a ratio of 4:1; the proportion of stones found in men reaching the age of 60 is more than 20% [4]. This preventable illness affects 7% to 13% of people in North America, 5% to 9% in Europe, and 1% to 5% in Asia [5]. Based on reports, the risk of illness development is 12% for men [6]. A 2019 study found that people had an inadequate understanding of diet-related risk factors for nephrolithiasis, but prior schooling improved respondents’ understanding of dietary risk factors. Participants were willing to adjust their diet if given the necessary knowledge. The significant recurrence incidence among those who formed stones highlights the need for extensive patient education on modifiable risk factors for nephrolithiasis [7]. According to a study conducted in Taif, the majority of participants were aware that their diet can affect the risk of kidney stones [8]. Health education initiatives should focus on dietary considerations for high-risk groups and modifiable risk factors, such as smoking and sedentary lifestyles, and be delivered through various media platforms [8]. In 2021, research conducted in the United Arab Emirates revealed that nearly fifty percent of the respondents were aware of different aspects of kidney stones, such as complications, diagnosis, and management. Individuals with a family history of kidney stones had significantly higher levels of knowledge of kidney stones than the other group. In addition, participants aged 40 to 49 years showed significantly higher levels of knowledge than the other groups. However, those with a higher education level did not necessarily have greater knowledge than the rest of the population, which was very enlightening [9]. This topic was chosen due to the high prevalence of kidney stones, the desire to investigate the impact of social media, and the limited number of published studies on this specific relationship. The aim of this study is to assess social media’s effect on public awareness towards dietary habits and fluid consumption on kidney stone formation.

## 2. Materials and Methods

### 2.1. Study Design

This observational cross-sectional study was carried out in Saudi Arabia from 1 April 2025 to 27 September 2025.

### 2.2. Sample Size

The sample size was calculated using Raosoft’s sample size calculator. Based on a 50% response distribution, a 5% margin of error, and a 95% confidence level, the required sample size was 384 participants. However, the sample size was further adjusted to account for the anticipated non-response rate. Assuming a 10% non-response rate, the final targeted sample size was determined to be 427 participants. Nevertheless, a total of 980 complete responses were obtained, which exceeds the minimum requirement and reduces the actual margin of error to approximately 3%.

### 2.3. Inclusion and Exclusion Criteria

Our inclusion criteria include adult individuals who are above 18 years old, who were Saudi and non-Saudi residing in Saudi Arabia, individuals who use social media platforms regularly (e.g., Instagram, Twitter/X, TikTok, Facebook, and Snapchat), individuals who agreed to participate in the study, and individuals who can read and understand Arabic. Our exclusion criteria include people under the age of eighteen, those who are outside Saudi Arabia or those who do not use social media, people who refuse to give their informed consent to take part in the research, people with no Arabic reading or comprehension skills, or people whose cognitive or psychological conditions make it difficult for them to understand or accurately complete the survey.

### 2.4. Method for Data Collection and Instruments (Data Collection Technique)

The study instrument was a structured, self-administered, and anonymous questionnaire in Arabic, adapted from a previous study [8]. The questionnaire (Supplementary Materials) was distributed through various social media platforms to ensure wide reach and participation. The Sample recruitment was conducted using social media websites (e.g., Facebook, Instagram, WhatsApp, Snapchat, Twitter) to reach people from all around Saudi Arabia. The survey included a combination of closed-response questions, open-response questions, and semantic differential scales to assess the impact of social media and digital information on public awareness of kidney health. It consisted of 23 questions arranged into five sections: general information, use of social media and health information sources, health behavior, kidney health awareness, and a general evaluation of the impact of social media. The data was collected using a non-probability sampling approach that combined convenience sampling for initial recruitment—which included direct outreach to known and unknown individuals, alongside an announcement on Telegram channels like Wamdha and others —with a snowball sampling technique, where participants were requested to share the survey with their eligible networks to maximize reach.

### 2.5. Pilot Study

The questionnaire was distributed to twenty respondents who were asked to complete it. This was carried out to evaluate the questionnaire’s clarity, simplicity, and feasibility for the study. The pilot study data were not included in the final analysis.

### 2.6. Scoring System

Participants’ knowledge and attitudes regarding kidney stone prevention and the role of social media were assessed using two separate scales. Knowledge was measured with six items, covering prevention methods and lifestyle or dietary factors related to kidney stone formation. Each correct answer was coded as 1, and incorrect or “I don’t know” responses as 0. One question allowed multiple correct responses, awarding one point per correct option. The total score, ranging from 0 to 9, was then converted into a percentage to facilitate interpretation and categorized according to Bloom’s cut-off points as poor (≤30%), moderate (31–59%), or good (≥60%). Attitudes were evaluated using four items reflecting participants’ beliefs, perceived positive and negative aspects of social media, and the reliability of online health information. One item used a five-point Likert scale, while the others were binary coded and recoded where necessary so that higher values indicated more positive attitudes. After standardizing all items, the total score ranged from 4 to 12. Preliminary reliability and factor analyses (Cronbach’s alpha and McDonald’s omega) confirmed that knowledge and attitude items represented district constructs. Although awareness was the overarching construct of interest, it was operationalized though these two dimensions (knowledge and attitudes) to capture both congestive and affective components of awareness. Therefore, each scale was analyzed separately in subsequent analyses.

### 2.7. Statistical Analysis

Data were cleaned and pre-processed using Microsoft Excel 365 (version 2508), and all statistical analyses were conducted using R version 4.5.1 (R Foundation for Statistical Computing, Vienna, Austria). Descriptive statistics were used to summarize demographic characteristics, with continuous variables presented with their means (±standard deviations), and categorical variables with their frequencies and percentages. Given the large sample size (>900), parametric tests were considered appropriate based on the central limit theorem. Multiple liner regression models were applied to identify factors associated with knowledge and attitudes toward kidney health, adjusting for theoretically relevant covariates including age, sex, and education level. The final models were selected based on adjusted R^2^ values and were overall statistically significant, and the results were presented using regression coefficients (β), standard error, and *p*-value. Finally, simple mediation analyses were performed to explore whether knowledge or attitudes mediated the relationship between social media use and kidney-health-related behaviors. The mediation models were considered exploratory and hypothesis-generating rather than causal. Bootstrap resampling (5000 samples) was used to estimate direct, indirect, and total effects with 95% confidence intervals. Statistical significance was set at *p* < 0.05.

## 3. Results

### 3.1. Demographic and Health Characteristics

A total of 980 individuals participated in this study, with an average age oF (29.9 ± 11) years, indicating that the majority of participants were young adults. The gender distribution of participants was 55.4% (543 males) and 44.6% (437 females). The results showed a clear disparity in educational levels, with the majority of participants, 69.9% (685 participants), holding university degrees, followed by middle or high school graduates at 20.9% (205 participants), those who had completed postgraduate studies at 8.2% (80 participants), and the lowest percentage, 1% (10 participants), representing those with primary education or lower. Regarding the health status of the participants, the vast majority, 88.9% (871 participants), confirmed that they did not suffer from any chronic diseases, while the remaining percentage was distributed among the following diseases: 7.1% (70 participants) suffered from high blood pressure, 5.8% (57 participants) suffered from diabetes, and the percentages were equal between heart and kidney disease at 0.9% (9 participants). Finally, 92.8% (909 participants) confirmed that they had not suffered from kidney stones before, while the remaining small percentage, 7.2% (only 71 participants), confirmed that they had suffered from kidney stones. All of these results are listed in Table 1 (Demographic and Health Characteristics of the Participants).

### 3.2. Knowledge Related to Kidney Health

Most participants showed a moderate level of knowledge about kidney health and kidney stone prevention (mean = 52.86, SD = ±21.89). About 44.5% of participants knew the recommended daily fluid intake for preventing kidney stones, and around 80% of respondents recognized that dietary habits can influence kidney health. In addition, 67% were aware that drinking enough water reduces the risk of kidney stones, while only 23.7% correctly identified that a high consumption of animal protein increases the risk. Regarding beverages, 73.2% of participants identified soft drinks as a risk factor, while fewer recognized the role of artificial juices (42.6%), alcohol (61.2%), and tea (11.9%). Furthermore, 71.3% believed that individuals who have had kidney stones are more likely to develop them again. Based on Bloom’s cut-off points, 30.3% of participants demonstrated good knowledge, 41.5% had a moderate level, and 28.2% showed poor knowledge. These results indicate that while general awareness of kidney health exists, there are still gaps in specific knowledge related to dietary- and beverage-related factors.

### 3.3. Attitudes Toward Kidney Health and Social Media

Most participants showed positive attitudes toward the role of social media in increasing awareness about kidney health (mean = 66.60, SD = ±14.95). Around 77% agreed or strongly agreed that social media helps improve public awareness, while 17.2% were neutral and 5.7% disagreed. For the positive aspects of social media, the majority (81.6%) believed that it allows easy access to health information. More than halF (58.8%) found the content to be simple and engaging, and 44.3% felt that it encourages healthy lifestyle changes. However, only 28.3% viewed the content as reliable. Regarding the negative aspects, 81.2% of participants mentioned the spread of misinformation, 67.7% pointed to conflicting opinions from non-experts, 50.5% noted difficulty in verifying information, and 45.7% believed that some content promotes unhealthy habits. When asked about the overall reliability of health information on social media, 66.5% answered “to some extent”, 19.5% said “no”, and only 14% considered it reliable. These findings suggest that participants appreciate social media as an accessible and engaging platform for health awareness, but many still question the accuracy and trustworthiness of the information provided.

### 3.4. Factors Associated with Knowledge and Attitudes Toward Kidney Health

Table 2 presents the results of the multiple linear regression models examining factors associated with knowledge and attitudes toward kidney health. The model for knowledge was statistically significant (F (14,965) = 13.46, *p* < 0.001) and explained approximately 15% of the variance in knowledge scores (Adjusted *R*^2^ = 0.151). Higher knowledge scores were significantly associated with older age (β = 0.18, *p* = 0.001), being female (β = −3.47, *p* = 0.009), relying on doctors or healthcare providers (β = 4.37, *p* = 0.003), and using medical websites as a main source of health information (β = 9.77, *p* < 0.001). Participants who followed health professionals on social media (β = 3.83, *p* = 0.013), watched health-related content on YouTube (β = 4.18, *p* = 0.005), or changed their dietary or fluid intake based on social media content (β = 6.57, *p* < 0.001) also had higher knowledge scores. In contrast, other sources such as family and friends or general social media use were not significantly associated with knowledge levels (*p* > 0.05).

The model for attitudes was also statistically significant (F (14,965) = 16.72, *p* < 0.001) and explained about 18% of the variance (adjusted *R*^2^ = 0.184). Significant predictors of positive attitudes included female sex (β = −3.97, *p* < 0.001), use of TikTok (β = 4.32, *p* < 0.001) or YouTube (β = 4.73, *p* < 0.001), and reliance on social media in general for health information (β = 3.04, *p* = 0.003). Participants who followed health professionals (β = 2.33, *p* = 0.019), accessed medical websites (β = 4.65, *p* < 0.001), or reported exposure to kidney health content (β = 1.46, *p* = 0.020) also demonstrated more positive attitudes. Other variables, including education level, family or friends, and not seeking health information, were not statistically significant predictors.

### 3.5. Mediation Analysis

#### 3.5.1. Mediation Analysis Using Knowledge as a Mediator

Table 3 presents the results of the mediation analysis examining the indirect role of knowledge in the relationship between exposure to digital health information sources and awareness of kidney health. The model showed that knowledge partially mediated the association between several information sources and awareness levels. Using medical websites had a significant total effect on awareness (β = 0.062, *p* = 0.020), mainly explained by an indirect path through knowledge (β = 0.040, *p* < 0.001), while the direct effect was not significant (*p* = 0.419). Similarly, YouTube did not show a significant total or direct association with awareness (*p* > 0.05), but a small yet significant indirect effect through knowledge was observed (β = 0.020, *p* < 0.001). In contrast, following health professionals on social media showed significant total (β = 0.334, *p* < 0.001) and direct effects (β = 0.310, *p* < 0.001), along with a smaller indirect effect through knowledge (β = 0.042, *p* < 0.001). Exposure to kidney health content also demonstrated significant total (β = 0.193, *p* < 0.001) and direct effects (β = 0178, *p* < 0.001), with a modest but significant indirect path through knowledge (β = 0.015, *p* < 0.001).

#### 3.5.2. Mediation Analysis Using Attitudes as a Mediator

The mediation analysis examined whether attitudes mediated the relationship between selected social media-related factors and kidney health awareness. As shown in Table 4, significant indirect effects were observed for all participants (*p* < 0.001). Attitudes significantly mediated the relationship between the use of medical websites and awareness (β = 0.035), even though the direct effect was not significant (*p* = 0.324). A similar pattern was found for YouTube use, where the indirect effect through attitudes was significant (β = 0.032) despite a non-significant direct path (*p* = 0.656). In contrast, both following health professionals on social media and exposure to kidney health content showed significant total, direct and indirect effects (all *p* < 0.001), suggesting that while attuites partially mediated these associations, direct effects remained strong.

## 4. Discussion

Renal stone disease is a widespread health issue that places considerable strain on both individuals and healthcare systems. It is a complex disorder influenced by food habits, fluid consumption, genetic predisposition, and environmental exposure. Recurrence rates are substantial, with as many as half of patients experiencing another episode within ten years if not taking preventive steps. Regarding the modifiable risk factors, eating habits and appropriate hydration have regularly been proven to play an important role in prevention [10].

Despite this information, public awareness is low in many areas, resulting in preventable morbidity. Public health tactics have historically focused on educational campaigns, but in the digital era, information-seeking behaviors have evolved towards online platforms, notably social media [11].

Recently, social media platforms (YouTube, TikTok, Twitter, Instagram, and Facebook) have been considered the main and easy sources of health information. Their unique advantages over traditional media include immediacy, interactive engagement, and wide accessibility across demographics. They also provide a venue for healthcare professionals, governmental organizations, and patient advocates to share reliable knowledge [12].

Unfortunately, there are also risks of misinformation, oversimplification, and unverified claims that may mislead the public. The dual role of social media as both a facilitator and a barrier to health literacy highlights the importance of examining its impact on specific health outcomes concerning possible hazards and malpractice [13].

In reference to renal stone prevention, where behavioral changes such as increasing water intake and reducing high-risk food components are necessary, the potential function of social media in influencing awareness justifies integrated attention and requires substantial research programs to gain a clear picture of the effect of social media on such an issue [14].

In the literature, a lot of previous research has examined the general awareness of kidney stone risk factors and dietary behaviors [15,16,17], but there remains a substantial gap in our understanding of how social media specifically influences public awareness and whether this translates into improved knowledge and behaviors.

In Saudi Arabia and other Middle Eastern countries, social media usage is among the highest globally, particularly among young adults [11,18]. However, there is limited evidence regarding its role in disseminating kidney-stone-related health information. This gap underscores the relevance of the present study, which tried to evaluate the effect of social media on dietary- and fluid-related awareness in kidney stone formation.

The aim of the current study was to find out how social media platforms affect public awareness of the connection between kidney stone formation and dietary practices and fluid intake. With an evaluation of the correlation between social media usage, demographic characteristics, and knowledge and attitude levels, the study sought to pinpoint the effects (advantages and disadvantages) of using social media as a tool for health promotion.

The study also assessed whether awareness was affected by following medical experts, viewing content from various platforms, and visiting medical websites, as well as whether demographic factors like age and gender moderated these effects.

The cross-sectional survey included 980 adult participants with diverse sociodemographic backgrounds. The mean age of the participants was 29.9%; this reflected a relatively young population, consistent with the high rate of social media use among this age group. The majority of respondents were male (55.4%), and a substantial proportion had university-level education.

Similarly, in a previous cross-sectional study using a self-administered questionnaire, distributed among 515 participants, the majority of participants were between 20 and 29 years old [9]. This was consistent with many previous studies that discussed this issue generally and not related to social media effects, where majority of participants was males in middle age [19,20].

The main obvious finding in the current study was that the analysis revealed that awareness levels regarding kidney stone prevention varied across domains. Subjects demonstrated good knowledge about the importance of water intake and the risks associated with excessive soft drink consumption.

Previous studies reported a lack of knowledge regarding appropriate water intake (46% had sufficient knowledge), followed by diet factors (only 50.1%) and risk factors of kidney stones (51.6%). All of these factors were combined to determine the total knowledge score, which represents the overall knowledge of the participants regarding each of the individual factors concerning kidney stones. The final mean of the total knowledge score was 56.4%, which surprisingly indicates a poor level of knowledge in the sample [9].

Another study enrolled a total of 1150 participants to assess the level of awareness of urolithiasis among the population of the Hail region in Saudi Arabia. Age ranged from 18 to 68 years, and the mean age was 26.3 ± 12.8 years old, with up to 51% being male. A total of 683 (59.4%) were found to have a low level of awareness regarding urolithiasis, 448 (39%) were moderately knowledgeable, while only 19 (1.7%) had a high awareness level [21].

Also, the level of awareness was fairly moderate regarding the recurrence of kidney stones and the need for consistent preventive measures and extensive educational programs. However, significant gaps existed in knowledge about the adverse effects of high animal protein consumption and tea intake, both of which are recognized dietary contributors to kidney stone risk, but unfortunately only a small percentage of participants knew this information.

Since protein-rich food consumption is a crucial factor in the pathogenesis of nephrolithiasis, some researchers have focused on dietary protein intake and its correlation with the incidence of kidney stones. Ingested protein as a rich source of purine, producing an acid load, increases urinary calcium and oxalate excretion, and seems to be involved in raising the risk of kidney stones [16,22,23].

In the study by Lv et al. [24], participants exhibited moderate attitudes and practices towards urinary stones, signaling a need for further enhancement, as seen in the knowledge dimension. For instance, the lowest average attitude score was for the item “Changing lifestyle and dietary habits to prevent urinary system stones brings about discomfort” (score: 2.84  ±  1.09). In terms of practices, the lowest score was for “My level of knowledge concerning urinary system stones is adequate to fulfill my requirements for stone prevention” (score: 2.35  ±  1.00). These responses indicate areas where public perception and behavior might be improved through targeted education and intervention [24].

In a previous systematic review, the authors showed that caffeine increases the urinary excretion of calcium, sodium and magnesium, in addition to having a diuretic action with consumption >300–360 mg (approximately four cups of coffee). Together with other components of coffee, this beverage might have potential protective effects against the formation of urinary stones. Tea exerts many protective effects against stone formation, through the accompanying water intake, the action of caffeine and the effects of components with antioxidant properties [25].

With regression analysis in the current study, we demonstrated that gender significantly influenced awareness, with males having lower awareness levels compared to females. Similarly, Bokhari et al. (2022) stated that the level of awareness about urolithiasis was studied in Abha, with the finding that females had higher levels of awareness than males [21].

Other studies have also found this higher level of awareness towards renal stones among females compared to males [20,26]. However, in contrast to the assumption that females have a higher level of awareness, Al Otipi et al. (2020) found that males displayed greater knowledge than females [27].

Another finding in our study was that age positively affected awareness, meaning that awareness increases with age. Similarly to this finding, previous studies concluded that older subjects had better awareness and knowledge about renal stones [24,28,29].

Importantly, the type of social media engagement mattered: those who followed medical professionals or accessed health-related content through YouTube and TikTok reported higher awareness. In contrast, reliance on unverified sources showed no meaningful benefit. Mediation analysis further revealed that knowledge and attitudes mediated the effect of social media use on awareness, suggesting that platforms may not only provide information but also shape attitudes toward preventive behaviors.

The influence of social media platforms themselves was considered a key insight. The finding that YouTube and TikTok users showed elevated awareness is consistent with the trend of increasing reliance on video-based content for health information. Videos can simplify complex medical advice, engage users emotionally, and facilitate retention [30].

However, the reliance on such platforms also still raises concerns regarding the quality and accuracy of content. Prior analyses of YouTube videos on kidney stones have shown that while some provide reliable information, a significant percentage include misleading or incomplete content with subsequent negative and undesirable effects [31]. Thus, the potential benefits observed in this study may reflect exposure to high-quality content but cannot guarantee accuracy without strict regulation and effective quality control.

The effects of following medical professionals on social media as a predictor of higher awareness aligns with earlier research suggesting that professional involvement enhances the use of online health information. This finding warrants the need to encourage more healthcare professionals to engage actively on digital platforms, where their expertise can counter misinformation and direct patients toward the right places for seeking medical and health advice [32].

In terms of methodology, the mediation effect of knowledge and attitudes is particularly noteworthy. This suggests that social media does not directly change behaviors but instead works indirectly by shaping understanding and perceptions. This is consistent with health behavior theories such as the Health Belief Model, which posits that knowledge and perceived susceptibility mediate the relationship between exposure to information and preventive behaviors. Thus, social media campaigns should not only disseminate facts but also address attitudes and beliefs to be effective [33,34].

The strengths of the current study lie in the relatively large sample size, the inclusion of diverse social media platforms, and the use of advanced statistical methods such as regression and mediation analyses, which helped identify both predictors and pathways of influence. These elements enhance the reliability and depth of the findings, particularly in highlighting how social media engagement—especially with healthcare professionals and video-based platforms—can positively influence knowledge and attitudes.

Nonetheless, some limitations should be acknowledged. The cross-sectional design prevents causal conclusions, and the reliance on self-reported data introduces risks of bias. The young and educated study population may also limit the generalizability of results to other groups, while the lack of assessment of the accuracy of social media content leaves questions about information quality unanswered.

## 5. Conclusions

This cross-sectional study clarifies the role of the digital environment in shaping both public knowledge and attitudes toward dietary and fluid-related factors associated with kidney stone formation. Our findings suggest that general awareness yields good results regarding basic aspects (such as hydration), but a persistent knowledge gap is revealed concerning more complex nutritional risk factors. Conversely, the results for attitudes demonstrate a significantly positive disposition toward consuming digital health information.

The key value of this study lies in applying regression and mediation analysis, which allowed us to explore the direct and indirect effects of information sources. The findings indicate that the efficacy of digital exposure is not exclusively direct but is likely mediated by improvements in both knowledge of and attitudes toward preventive behavior.

Source credibility emerged as a primary driver of the effects discovered. Reliance on official medical websites was the strongest predictor for increased knowledge, while following health professionals on visual platforms correlated with a positive surge in both attitudes and knowledge. This confirms that social media is a valuable channel for health education when used to deliver expert-led, credible information.

Therefore, public health protocols should integrate expert-driven educational strategies to address complex knowledge deficiencies. We recommend that future longitudinal studies evaluate content accuracy and conclusively verify whether these discovered effects translate into sustained, measurable preventive behavioral change.

### Recommendations

Future longitudinal and qualitative studies, alongside regulatory efforts, are recommended to ensure the accurate dissemination of health information and confirm whether greater awareness leads to lasting behavioral change.

## Figures and Tables

**Table 1:** Demographic and health characteristics of the participants.

Characteristic		n (N = 980) or (Mean, SD)	Percentage (%)
Age		(29.9 ± 11)	
Sex	Female	437	(44.6%)
	Male	543	(55.4%)
Educational Level	Primary or lower	10	(1%)
	Middle/High School	205	(20.9%)
	University	685	(69.9%)
	Postgraduate	80	(8.2%)
Do you suffer from any of the following chronic conditions?	High blood pressure	70	(7.1%)
	Diabetes	57	(5.8%)
	Heart disease	9	(0.9%)
	Kidney disease	9	(0.9%)
	None	871	(88.9%)
Have you ever had kidney stones?	No	909	(92.8%)
	Yes	71	(7.2%)

**Table 2:** Multiple liner regression analysis of factors associated with knowledge and attitudes toward kidney health.

Variable	Knowledge		Attitudes	
	β (SE)	*p*-Value	β (SE)	*p*-Value
Intercept	25.35 (5.35)	2.48 × 10^−6^	51.72 (3.72)	<2.2 × 10^−16^
Demographics				
Age	0.18 (0.05)	0.0015	0.005 (0.03)	0.8848
Sex (Male)	−3.47 (1.33)	0.0095	−3.97 (0.89)	8.946 × 10^−6^
Educational Level	2.14 (1.16)	0.0664	0.77 (0.80)	0.3387
Health information source				
Social media	1.33 (1.50)	0.3759	3.04 (1.01)	0.0027
Doctor/Healthcare provider	4.37 (1.49)	0.0035	2.30 (0.99)	0.0203
Medical websites	9.77 (1.37)	1.92 × 10^−12^	4.65 (0.88)	2.052 × 10^−7^
Family/friends	−2.94 (1.69)	0.0816	−0.27 (1.17)	0.8138
No health information	−2.79 (2.95)	0.3443	−2.92 (2.11)	0.1678
Platform used				
TikTok	2.46 (1.53)	0.1090	4.32 (1.00)	1.977 × 10^−5^
YouTube	4.18 (1.48)	0.0048	4.73 (0.97)	1.553 × 10^−6^
No social media use (for health content)	2.26 (1.99)	0.2556	4.32 (1.36)	0.0015
Social media-related behaviors				
Follow health professionals on social media	3.83 (1.53)	0.0126	2.33 (0.99)	0.0186
Exposure to kidney health content	1.35 (0.87)	0.1195	1.46 (0.63)	0.0203
Diet/fluid intake based on social media	6.57 (1.70)	0.0001	5.05 (1.12)	8.288 × 10^−6^

Note. Regression coefficients are reported with heteroskedasticity robust standard errors (HC1 correction). Significant predictors are shown at *p* < 0.05.

**Table 3:** Mediation analysis using knowledge as a mediator.

Variable	Total Effects		Direct Effects		Indirect Effect	
	β (95% CI)	*p*-Value	β (95% CI)	*p*-Value	β (95% CI)	*p*-Value
Medical websites	0.062 (0.010, 0.115)	0.020	0.022 (−0.031, 0.077)	0.419	0.040 (0.024, 0.057)	<0.001
YouTube	0.021 (−0.031, 0.076)	0.437	0.0007 (−0.051, 0.055)	0.984	0.020 (0.009, 0.033)	<0.001
Following health professionals on social media	0.334 (0.275, 0.393)	<0.001	0.310 (0.250, 0.368)	<0.001	0.024 (0.012, 0.039)	<0.001
Exposure to kidney health content	0.193 (0.161, 0.214)	<0.001	0.178 (0.143, 0.202)	<0.001	0.015 (0.007, 0.025)	<0.001

**Table 4:** Mediation analysis using attitudes as a mediator.

Variable	Total Effects		Direct Effects		Indirect Effect	
	β (95% CI)	*p*-Value	β (95% CI)	*p*-Value	β (95% CI)	*p*-Value
Medical websites	0.062 (0.009, 0.115)	0.0208	0.027 (−0.025, 0.080)	0.3240	0.035 (0.021, 0.052)	<0.001
YouTube	0.020 (−0.032, 0.074)	0.4592	−0.011 (−0.064, 0.042)	0.6564	0.032 (0.018, 0.047)	<0.001
Following health professionals on social media	0.334 (0.276, 0.393)	<0.001	0.304 (0.245, 0.362)	<0.001	0.031 (0.017, 0.047)	<0.001
Exposure to kidney health content	0.194 (0.162, 0.215)	<0.001	0.173 (0.138, 0.196)	<0.001	0.021 (0.011, 0.032)	<0.001

## Data Availability

The data that support the findings of this study are available from the corresponding author upon reasonable request. The data are not publicly available due to ethical restrictions, as they contain information that could compromise the privacy of research participants.

## Supplementary Materials

The following supporting information can be downloaded at: https://www.mdpi.com/article/10.3390/healthcare13212795/s1.
