# Supplementary material for: Social Media’s Impact on Public Awareness of the Effects of Dietary Habits and Fluid Consumption on Kidney Stone Formation: A Cross-Sectional Study
Source: Healthcare (Basel). 2025 Nov 4;13(21):2795. doi: 10.3390/healthcare13212795 (PMC12607395; doi:10.3390/healthcare13212795)
Supplement: Supplementary file 1 [file healthcare-13-02795-s001.zip › healthcare-3927743-supplementary.pdf]

أثر وسائل التواصل والمعلومات الرقمية على وعي المجتمع بصحة الكلى

عزيزي/عزيزتي المشارك/ة،

شكرًا لك على وقتك في إكمال هذا الاستبيان، سوف يستغرق من 3 إلى 5 دقائق لإكماله.

نحن طلاب طب من جامعات مختلفة في المملكة العربية السعودية، نقوم بإجراء دراسة بحثية تهدف إلى م
عرفة مدى وعي
المجتمع بصحة الكلى والعوامل الخطرة المرتبطة بها، ودور وسائل التواصل والمعلومات الرقمية في تعز
يز هذا الوعي بين سكان المملكة العربية السعودية.

ستظل بياناتك مجهولة الهوية وتُستخدم لأغراض البحث العلمي فقط، ولن يتم تحديد هوية المشاركين بالأ
سماء.

نقدّر إجابتك الصادقة.

هل توافق على المشاركة؟

- نعم
- لا

المعلومات العامة:

1. العمر
2. الجنس
   - ذكر
   - أنثى
3. المستوى التعليمي
   - ابتدائي أو أقل
   - متوسط / ثانوي
   - جامعي
   - دراسات عليا
4. هل تعاني من أي من الأمراض التالية؟
   - ارتفاع ضغط الدم
   - السكري

- أمراض القلب
- أمراض الكلى
- لا أعاني من أمراض مزمنة

استخدام وسائل التواصل الاجتماعي ومصادر المعلومات الصحية:

5. ما هو المصدر الأساسي الذي تعتمد عليه للحصول على معلوماتك الصحية؟
   - وسائل التواصل الاجتماعي
   - طبيب أو مقدم رعاية
   - مواقع إلكترونية طبية
   - العائلة أو الأصدقاء
   - لا أبحث عن معلومات صحية
6. كم ساعة تقضي يوميًا على وسائل التواصل الاجتماعي؟
   - أقل من ساعة
   - 1–3 ساعات
   - 4–6 ساعات
   - أكثر من 6 ساعات
7. ما المنصات التي تستخدمها غالبًا للمحتوى الصحي؟ (يمكن اختيار أكثر من خيار)
   - إنستغرام
   - تيك توك
   - تويتر
   - فيسبوك
   - يوتيوب
   - لا أستخدم وسائل التواصل في المجال الصحي
8. هل تتابع مختصين صحيين (مثل أطباء أو أخصائيي تغذية) على وسائل التواصل؟
   - نعم
   - لا
9. كم مرة تشاهد محتوى متعلق بصحة الكلى أو التغذية أو شرب الماء على وسائل التواصل؟
   - بشكل متكرر جداً
   - أحياناً
   - نادراً
   - أبداً
10. هل سبق أن غيّرت سلوكك الغذائي أو استهلاكك للسوائل بناءً على ما شاهدته في وسائل التواصل؟
    - نعم
    - لا

السلوك الصحي والوعي بصحة الكلى:

11. كم لترًا من الماء تشرب يوميًا تقريبًا؟

- أقل من لتر
- من ١ إلى ٢ لتر
- من ٢ إلى ٣ لتر
- أكثر من ٣

12. هل تعرف الكمية اليومية الموصى بها من السوائل للوقاية من تكوّن حصى الكلى؟

- نعم
- لا
- غير متأكد

13. هل تعتقد أن العادات الغذائية تؤثر على صحة الكلى وتكوّن الحصوات؟

- نعم
- لا
- غير متأكد

14. كيف يؤثر شرب الماء على خطر الإصابة بحصى الكلى؟

- يزيد
- يقلل
- لا يؤثر
- لا أعلم

15. كيف يؤثر البروتين الحيواني على خطر الإصابة بحصى الكلى؟

- يزيد
- يقلل
- لا يؤثر
- لا أعلم

16. ما المشروبات التي تعتقد أنها تزيد من خطر الإصابة بحصى الكلى؟ (يمكن اختيار أكثر من خيار)

- المشروبات الغازية
- القهوة
- العصائر الصناعية
- الكحول
- الشاي
- الماء
- لا أعلم

17. هل سبق أن أُصبت بحصى الكلى؟

- نعم
- لا

18. هل تعتقد أن الأشخاص الذين أصيبوا بحصى الكلى أكثر عرضة لتكرار الإصابة؟
- نعم
- لا

التقييم العام لتأثير وسائل التواصل على الوعي الصحي:

19. هل ترى أن وسائل التواصل الاجتماعي تساعد على زيادة وعي الناس بصحة الكلى؟
- أوافق بشدة
- أوافق
- محايد
- لا أوافق
- لا أوافق بشدة
20. في رأيك، ماالجوانب الايجابية في استخدام وسائل التواصل في التوعية الصحية؟(يمكن اختيار أكثر من خيار)
- سهولة الوصول للمعلومة
- أسلوب مبسط وشيق
- محتوى موثوق
- تحفيز على تغيير نمط الحياة
- لاتوجد ايجابيات
21. في رأيك، ما الجوانب السلبية لاستخدام وسائل التواصل في التوعية الصحية ؟ (يمكن اختيار اكثر من خيار)
- انتشار معلومات خاطئة
- تضارب أراء غير مختصين
- صعوبة التحقق من المعلومة
- الترويج لعادات غير صحية
- لا توجد سلبية
22. هل تعتقد أن وسائل التواصل توفر معلومات صحية دقيقة وموثوقة؟
- نعم
- لا
- إلى حداً ما

Social Media's Effect on Public Awareness Towards Dietary Habits and Fluid Consumption on Kidney Stone Formation

Do you agree to participate in this study?

- Yes
- No

A. Personal Information:

1) **Age**
2) **Gender**
    - Male
    - Female
3) **Educational Level**
    - Elementary School or below
    - Secondary/Intermediate or High School
    - Undergraduate (Bachelor's degree)
    - Postgraduate (Master's or Doctorate)
4) **Do you have any of the following medical conditions?**
    - Hypertension
    - Diabetes
    - Heart Disease
    - Kidney Disease
    - I don't have a medical condition

B. Social media usage and sources of health information:

5) **What is your main source for health information?**
    - Social media
    - Physicians or Healthcare Providers
    - Medical Websites
    - Family and Friends
    - I don't look for health information
6) **How many hours do you spend on social media per day?**

- Less than an hour a day
- 1-3 hours a day
- 4-6 hours a day
- More than 6 hours a day

7) **Which of the following platforms do you mostly use to obtain your health-related information?** (you may select more than one)
   - Instagram
   - TikTok
   - Twitter/X
   - Facebook
   - YouTube
   - I don't use social media for health-related information
8) **Do you follow healthcare providers (e.g., doctors or dietitians) on social media?**
   - Yes
   - No
9) **How often do you engage with content related to preventing kidney diseases, nutrition, or water intake?**
   - Always
   - Sometimes
   - Rarely
   - Never
10) **Have you ever changed your dietary habits or fluid intake based on information from on social media?**
   - Yes
   - No

C. Knowledge, Attitudes, and Behaviors Regarding Kidney Health:

11) **Approximately how many liters of water do you drink daily?**
   - Less than 1 liter per day

- Between 1 to 2 Liters per day
- Between 2 to 3 Liters per day
- More than 3 Liters per day

12) **Do you know the recommended daily fluid intake to prevent the formation of kidney stones?**

- Yes
- No
- Not Sure

13) **Do you think dietary habits affect kidney and stones formation?**

- Yes
- No
- Not Sure

14) **How does water intake affect the risk of kidney stone formation?**

- Water intake increases the risk
- Water intake decreases the risk
- Water intake doesn't affect the stone formation
- I don't know

15) **How do animal proteins affect the risk of kidney stone formation?**

- Animal proteins increase the risk
- Animal proteins decrease the risk
- Animal proteins don't affect the stone formation
- I don't know

16) **Which of the following drinks do they think are most likely increase risk of kidney stone formation?** (you may select more than one)

- Soft Drinks
- Coffee
- Artificial Juices

- Alcohol
- Tea
- Water
- I don’t know

17) Have you ever had kidney stones?

- Yes
- No

18) Do you think people who developed kidney stones before are more likely to develop them again?

- Yes
- No

D. Assessment of effect of Social Media on Public Awareness:

19) Do you agree that social media enhances public awareness on preventing kidney diseases and stones formation?

- Strongly Agree
- Agree
- Neutral
- Disagree
- Strongly Disagree

20) In your opinion, what are the advantages of using social media to raise public awareness? (you may select more than one)

- Easy access to information
- Simple and engaging delivery
- Reliable source
- Promote healthy lifestyle
- No Advantages

21) In your opinion, what are the disadvantages of using social media to raise public awareness? (you may select more than one)

- Easy spread of misinformation

- Conflicting opinions from non-specialists
- Difficulty of verifying information
- Promoting unhealthy lifestyle
- No Disadvantages

22) Do you think social media provides accurate and reliable health information?

- Yes
- No
- To some extent
